# FGF2-Derived PeptibodyF2-MMAE Conjugate for Targeted Delivery of Cytotoxic Drugs into Cancer Cells Overexpressing FGFR1

**DOI:** 10.3390/cancers12102992

**Published:** 2020-10-15

**Authors:** Karolina Jendryczko, Julia Chudzian, Natalia Skinder, Łukasz Opaliński, Jakub Rzeszótko, Antoni Wiedlocha, Jacek Otlewski, Anna Szlachcic

**Affiliations:** 1 Department of Protein Engineering, Faculty of Biotechnology, University of Wroclaw, 50383 Wroclaw, Poland; karolina.jendryczko@uwr.edu.pl (K.J.); julia.chudzian@uwr.edu.pl (J.C.); skinder.natalia@gmail.com (N.S.); lukasz.opalinski@uwr.edu.pl (Ł.O.); 292762@uwr.edu.pl (J.R.); jacek.otlewski@uwr.edu.pl (J.O.); 2 Department of Molecular Cell Biology, Institute for Cancer Research, The Norwegian Radium Hospital, Oslo University Hospital, 0379 Oslo, Norway; Antoni.Wiedlocha@rr-research.no; 3 Department of Radiobiology and Radiation Protection, Military Institute of Hygiene and Epidemiology, 01163 Warsaw, Poland; 4 Center for Cancer Cell Reprogramming, Institute of Clinical Medicine, Faculty of Medicine, University of Oslo, 0379 Oslo, Norway

**Keywords:** peptibody, targeted cancer therapy, FGFR, FGF2, protein–drug conjugate

## Abstract

**Simple Summary:**

Current approaches to treat cancer include eg. targeted therapies, employing agents, such as antibodies, aimed directly at molecules expressed in cancerous cells. Here we present a targeting molecule alternative to antibodies-peptibodyF2-composed of a peptide responsible for its targeting properties, and an immunoglobulin fragment increasing its stability and improving pharmacokinetic properties. Peptibody F2 specifically binds to fibroblast growth factor receptors (FGFRs) upregulated in multiple types of cancers. Moreover, peptibodyF2 conjugated with the cytotoxic drug (MMAE) serves as an efficient and selective drug carrier delivering cytotoxic payload to cancerous cells expressing FGFR1, leading to their death.

**Abstract:**

Fibroblast growth factor receptors (FGFRs) are emerging targets for directed cancer therapy. Presented here is a new FGFR1-targeting conjugate, the peptibodyF2, which employs peptibody, a fusion of peptide and the Fc fragment of human IgG as a selective targeting agent and drug carrier. Short peptide based on FGF2 sequence was used to construct a FGFR1-targeting peptibody. We have shown that this peptide ensures specific delivery of peptibodyF2 into FGFR1-expressing cells. In order to use peptibodyF2 as a delivery vehicle for cytotoxic drugs, we have conjugated it with MMAE, a drug widely used in antibody–drug conjugates for targeted therapy. Resulting conjugate shows high and specific cytotoxicity towards FGFR1-positive cells, i.e., squamous cell lung carcinoma NCI-H520, while remaining non-toxic for FGFR1-negative cells. Such peptibody–drug conjugate can serve as a basis for development of therapy for tumors with overexpressed or malfunctioning FGFRs.

## 1. Introduction

Although systemic chemotherapy still remains the main treatment strategy for cancer, progress in the field of targeted therapeutics and cancer diagnostics allowed for the rapid development of personalized medicine. Multiple cancer markers have been identified to distinguish between cancerous and normal cells and molecular profiling of patients is becoming more common [1]. Targeted therapy is advantageous because of its specific interference with cancer cells’ characteristics and thus reduced toxicity towards healthy tissues resulting in less severe side effects [2].

Growth factors and their receptors are widely investigated as molecular targets in anticancer therapy. Studies show that their aberrations are involved in growth and progression of different malignancies [3,4,5]. Fibroblast growth factor receptors (FGFRs) are cell surface proteins that in conjunction with their ligands, fibroblast growth factors (FGFs), transduce mitogenic and prosurvival signals through the plasma membrane. FGFRs are overexpressed in various types of cancers (e.g., lung, breast and prostate cancers), and are involved in pathogenesis, tumor growth and progression [6,7]. Overexpression of FGFR1 is one of the most frequent genetic aberrancies within the FGFR family in cancers [8,9,10].

Until now, there are two approved FGFR-targeted therapeutics for cancer treatment, both small molecule kinase inhibitors: erdafinitib, for the treatment of FGFR2- or FGFR3-expressing bladder cancers [11], and pemigatinib, for advanced bile duct cancer [12]. High hopes rest on FGFR-targeting therapies based on monoclonal antibodies, with multiple candidates being studied in clinical trials. Here, we explore an FGFR-targeting agent based on a peptide-Fc fusion, peptibodyF2, a targeting molecule alternative to antibodies. Even though monoclonal antibodies offer high binding specificity and strength, good pharmacokinetic profile and the ability to induce an immune response by directing immune cells to the place where antibody was bound [13,14,15], peptides fused with IgG Fc fragment share some of these characteristics while retaining the ease of unnatural/modified amino acids incorporation and smaller size compared to antibodies [16,17]. The design of new peptibodies can be also facilitated by the very large number of peptides characterized up to date with regard to their binding properties and ability to disrupt certain cellular processes. Peptibodies can be also easily produced at large scale in bacteria expression systems [18,19,20]. These qualities make peptibodies a promising alternative for monoclonal antibodies, resulting in many of them being tested in different stages of preclinical and clinical development, with two peptibody drugs (Romiplostim and Dulaglutide) approved and available on the market [21,22].

In this study, we describe a peptibody binding to FGFR1 present on the cell surface, named peptibodyF2. As a targeting portion of the peptibodyF2 we used a combination of two FGF2-derived peptides identified before as FGFR1-binders [23,24]. To ensure the therapeutic potency, we have conjugated the peptibodyF2 with monomethyl auristatin E (MMAE), a cytotoxic drug well characterized for its clinical application in a conjugate format in antibody–drug conjugates (ADCs).

We show that peptibodyF2 utilizes receptor-mediated endocytosis for internalization into FGFR1-expressing cells, into both engineered (transfected with ***fgfr1*** gene) and lung cancer cell lines with elevated levels of FGFR1 expression. Furthermore, we demonstrate that peptibodyF2 serves as an efficient and selective drug carrier as it delivers MMAE to FGFR1 expressing cells leading to their death with little effect on FGFR1-negative cells.

## 2. Results

### 2.1. Design, Expression and Purification of FGFR-Targeting Peptibody

**Figure 1:**
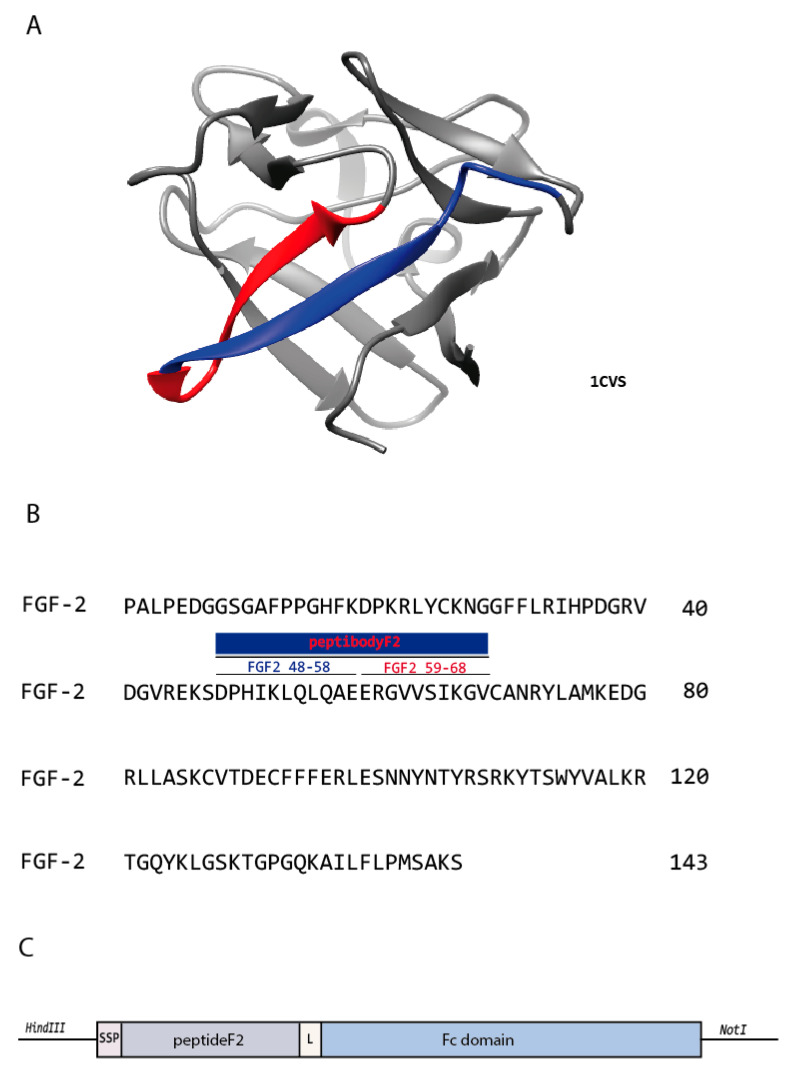
Design and sequence of the fibroblast growth factor receptor (FGFR)-targeting peptibody. (**A**) Structure of FGF2 (grey, PDB ID:1CVS) with sequences 48–58 and 59–68 depicted in blue and red, respectively. (**B**) FGF2 sequence with marked amino acid sequences constituting the peptideF2. (**C**) Genetic construct of peptibodyF2 in pLEV113 plasmid; SP—signal peptide; L—linker.

One of the benefits of peptibody design is the ability to take advantage of all the information available for previously characterized peptide sequences. Among the peptides identified so far as potential FGFR-binders, either by rational design or phage display selections, two peptides corresponding to FGF2 sequence regions: DPHIKLQLQAE, FGF2 residues 48–58 [23] and ERGVVSIKGVCA, FGF2 residues 59–68 [24] have been independently identified (Figure 1). Moreover, peptide DPHIKLQLQAE was described in the literature as a potent agonist of fibroblast growth factor receptor 1 (FGFR1) [23].

Based on these observations we designed peptibodyF2—a peptideF2 fused at the C-terminus to the Fc fragment of human IgG. PeptideF2 is spanning over residues 48–68 from the FGF2 sequence. Considering the fact that these two sequences are located directly next to each other in the linear structure of the growth factor, we decided to combine these two peptides into one to maximize the region interacting with FGFR1 (Figure 1). Such sequence may potentially benefit from the combination of two adjacent FGFR1 binding sites and exhibit higher affinity compared to shorter peptides described before, as short peptidic binders suffer from relatively low binding affinities resulting from, e.g., entropic effects. Moreover, a glycine-serine linker (GGSGG) was introduced between the Fc fragment and peptide F2 to ensure flexibility.

**Figure 2:**
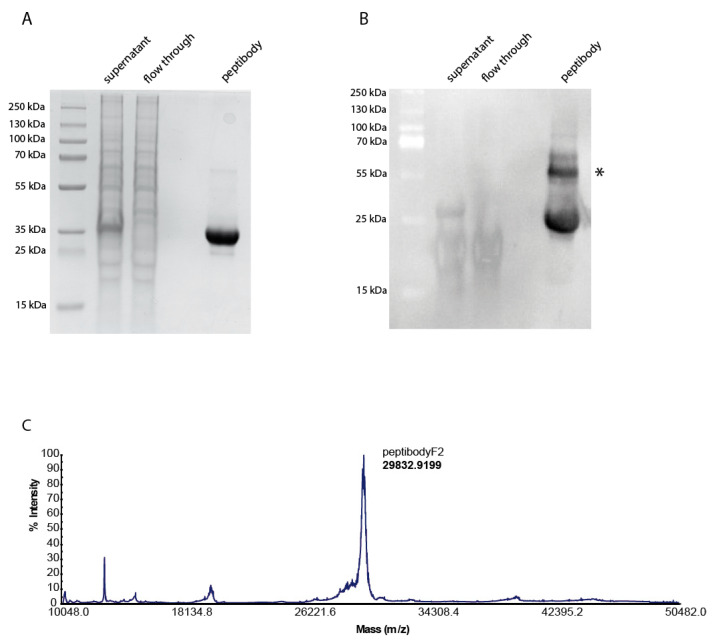
High yield expression and purification of peptibodyF2 from CHO cells. CHO cells were trasfected with peptibodyF2 in pLEV113 vector with signal peptide to facilitate the export of recombinant protein to the medium. SDS-PAGE followed by Coomassie staining (**A**) and anti-Fc Western blot (**B**) analysis of peptibodyF2 ProteinA-affinity purification process. Due to a not complete sample reduction small amounts of recombinant protein dimer (*) can be detected by Western blot. (**C**) Correct molecular weight of the peptibody as verified by MALDI-MS.

To provide proper folding and correct glycosylation pattern of Fc domain in the recombinant protein, the construct was expressed in CHO cells based on a protocol previously developed in our group, with the use of N-terminal signal peptide facilitating export of recombinant protein to the medium and subsequent affinity purification on immobilized ProteinA [25]. More than 50 mg of at least 95% pure peptibodyF2 was obtained using this procedure as shown with SDS-PAGE and confirmed by mass spectrometry (Figure 2).

### 2.2. PeptibodyF2 Binds FGFR1 and Induces Receptor Activation, but Not Cell Proliferation

**Figure 3:**
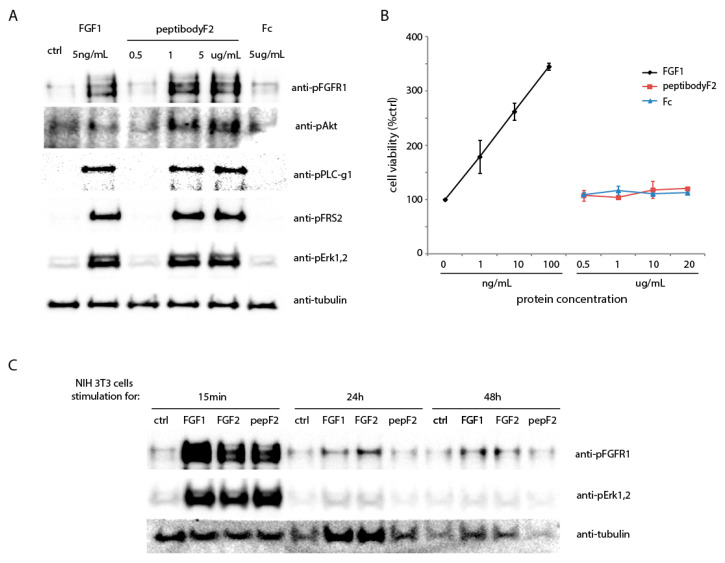
Activation of FGFR-dependent signaling pathways in different cell lines analyzed by Western blot. (**A**) Phosphorylation of FGFR1 and its downstream kinases in NIH 3T3 cells after incubation with increasing concentrations of peptibodyF2. (**B**) Proliferative response of NIH 3T3 cells treated for 48 h with peptibodyF2, Fc alone or FGF1. All experiments were normalized to the values for non-treated cells recognized as 100%. The error bars represent ± SD for ***n*** = 3 experiments. (**C**) PeptibodyF2-induced activation of FGFR is not sustained for prolonged periods of time. Serum-starved NIH 3T3 cells were stimulated with FGF1, FGF2 or peptibodyF2 for 15 min, 24 h or 48 h at 37 degrees, lysed and analyzed for FGFR1 and Erk1,2 phosphorylation.

Since one of the peptides used for peptibodyF2 construction was characterized before as the FGF2 agonist, we decided to evaluate the peptibodyF2 ability to bind to FGFR1 present on the cell surface and induce receptor-dependent signaling pathways. We applied mouse embryo fibroblast cells - NIH 3T3, routinely used for FGF-induced stimulation assays. After stimulation with either peptibodyF2 or FGF1, phosphorylated FGFR1 and receptor downstream kinases were detected by Western blotting (Figure 3A).

As shown in Figure 3A, peptibodyF2 caused efficient phosphorylation of FGF receptors and receptor-downstream kinases. Levels of phosphorylation of Akt kinase, FRS2 and phospholipase C-gamma 1 are increased upon stimulation with FGF1, as expected, and an increase in phosphorylation signal was observed for concentrations of peptibodyF2 above 1 μg/mL. Noteworthy, the concentrations of peptibodyF2 required to activate FGFR-downstream signaling were much higher than for natural FGFR1 ligand, FGF1. Fc fragment alone did not cause any effects in all tested concentrations. These data demonstrated that peptibodyF2 binds to FGFR1 present on the cell surface, leading to receptor activation.

FGFR1 stimulation leads to cell division, provided that the receptor activation is lasting. To verify if the residual receptor stimulation by peptibodyF2 can lead to proliferative effects, we treated NIH 3T3 cells with peptibodyF2, Fc alone or FGF1 and measured cell viability (Figure 3B). As expected, FGF1 caused a significant proliferative effect. However, peptibodyF2 did not stimulate cell division. In order to check if its binding to the receptor is too transient to induce a long-term cellular response, we analyzed the activation of FGFR at longer timepoints (24 h and 48 h, Figure 3C). PeptibodyF2 was unable to activate FGFR signaling for longer periods of time, which is in line with our interpretation. Significant differences in detected tubulin levels (selected loading control) can be observed for 48 h stimulation sample, but culturing NIH 3T3 cells for 48 h in serum-deprived medium may lead to their decreased viability if no growth factors are present.

### 2.3. PeptibodyF2 Is Specifically Internalized by FGFR-Expressing Cells

**Figure 4:**
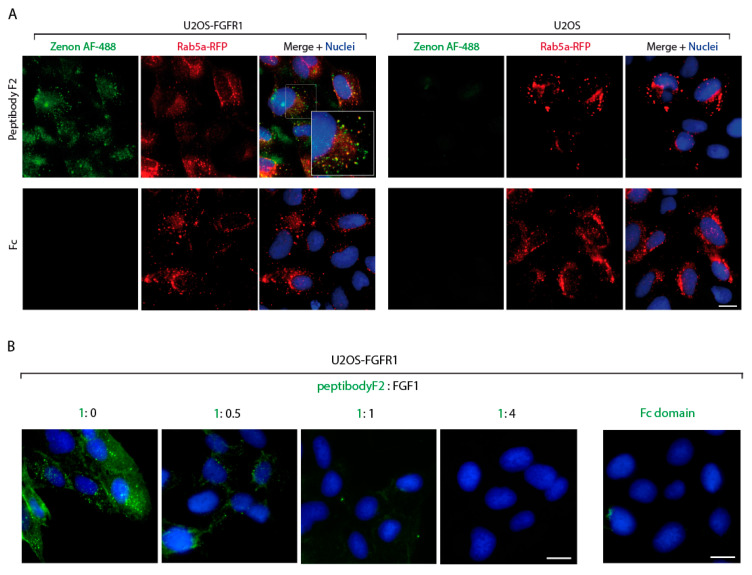
PeptibodyF2 is specifically internalized by FGFR-expressing cells. (**A**) U2OS stably transfected with FGFR1 were incubated with peptibodyF2 and Fc domain. All proteins were labeled with Zenon-488 dye and nuclei were fluorescently labeled with NucBlue. (**B**) Evaluation of competitive-binding of the FGFR-targeted peptibody. Increasing concentrations of unlabeled FGF1 (natural FGFR1 ligand) and peptibody—Zenon 488 were added. NucBlue was added for nuclei visualization. Scale bar equals to 20 μm.

The FGF1-induced dimerization and activation of FGFR1 triggers receptor endocytosis leading to the attenuation of signal transduction. We used fluorescence microscopy to study the internalization of peptibodyF2. As shown in Figure 4A peptibodyF2-derived signal was observed in U2OS-FGFR1 cells as numerous spots that largely colocalized with an early endosome marker Rab5a. In contrast, no fluorescence of peptibodyF2 was detected in U2OS cells devoid of FGFR1 (Figure 4A). The intracellular signal for the Fc fragment alone was undetectable in both studied cell lines (Figure 4A).

PeptibodyF2 consists of FGF-derived sequence responsible for the interaction with FGFRs. To confirm that the cellular uptake of peptibodyF2 is a result of the peptibodyF2 binding to the ligand recognition region within FGFR1 we performed competitive internalization studies with non-labeled FGF1. At a 1:1 ratio of FGF1 to peptibodyF2 we observed only residual intracellular fluorescence of peptibodyF2, and at a 4:1 ratio FGF1 no peptibodyF2 signal was detected in U2OS-FGFR1 cells (Figure 4B).

These data demonstrate that it was the peptideF2 sequence within peptibodyF2 that facilitated binding of the peptibodyF2 to FGFR1 present on the cell surface. Subsequently, peptibodyF2 was selectively taken up by the cells via FGFR1-dependent endocytic processes.

### 2.4. Site-Specific Conjugation of Monomethyl Auristatin E to the PeptibodyF2

**Figure 5:**
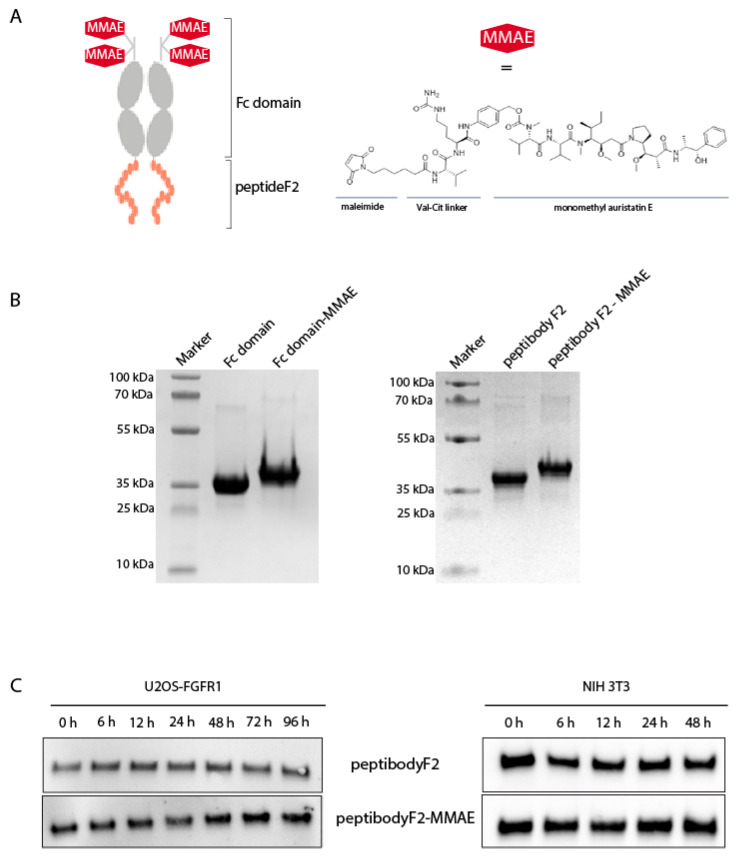
PeptibodyF2 efficiently conjugated with monomethyl auristatin E. (**A**) Scheme of peptibodyF2-MMAE conjugate. (**B**) SDS-PAGE electrophoresis followed by Coomassie staining used to visualize conjugation reaction product for peptibodyF2 and Fc domain with MMAE. (**C**) Amount of peptibodyF2 or peptibodyF2-MMAE conjugate present in the medium above U2OS-FGFR1 and NIH 3T3 cells. Proteins have been visualized by Western blotting with anti-Fc antibodies.

Taking advantage of the FGFR1-binding properties of peptibodyF2 and its ability to enter the cells strictly via FGFR1-dependent endocytosis, we decided to use it as a cytotoxic drug carrier, similarly as antibodies in antibody–drug conjugates (ADCs). Monomethyl auristatin E was a drug of our choice, as it has become a gold standard for many conjugate preclinical developments. The attachment of MMAE to peptibodyF2 was conducted via maleimidethiol modification of TCEP-reduced cysteine residues naturally occurring in the hinge region of the Fc fragment, in like manner to modifications of antibodies with cytotoxic drugs [26,27] (Figure 5A). Similarly to FDA-approved antibody–drug conjugates, a cleavable valine-citrulline linker was used, to ensure cytotoxic payload release only after entering the cell. The valine-citrulline linker was shown to be cleaved by cathepsin B in the lysosomal compartment, leading to drug release and inhibition of tubulin polymerization [28,29]. As a specificity control we prepared the MMAE conjugate of the Fc fragment alone. The efficiency of conjugation was very high (>99%), as no unmodified peptibodyF2 or Fc fragment were detected after a completed conjugation reaction (as estimated by SDS-PAGE and Coomassie staining). To remove any remaining free MMAE, the conjugates were purified on Protein A—Sepharose resin, additionally confirming that there was no unfolding of the Fc domain caused by the drug attachment (Figure 5B).

We conducted stability analysis of both peptibodyF2 and its MMAE-conjugate in the cell-conditioned medium. We used NIH 3T3 cells and U2OS-FGFR1 cells, and no significant changes in the amount of peptibodyF2 or its conjugate in the cell-conditioned medium were observed (Figure 5C). Furthermore, we did not observe any shift in protein migration profile in SDS-PAGE, an indication of spontaneous release of MMAE from the peptibody. Therefore, we concluded that peptibodyF2 was stable and free MMAE was not released in the time course of the experiments.

### 2.5. Cytotoxicity of PeptibodyF2-MMAE Conjugates

**Figure 6:**
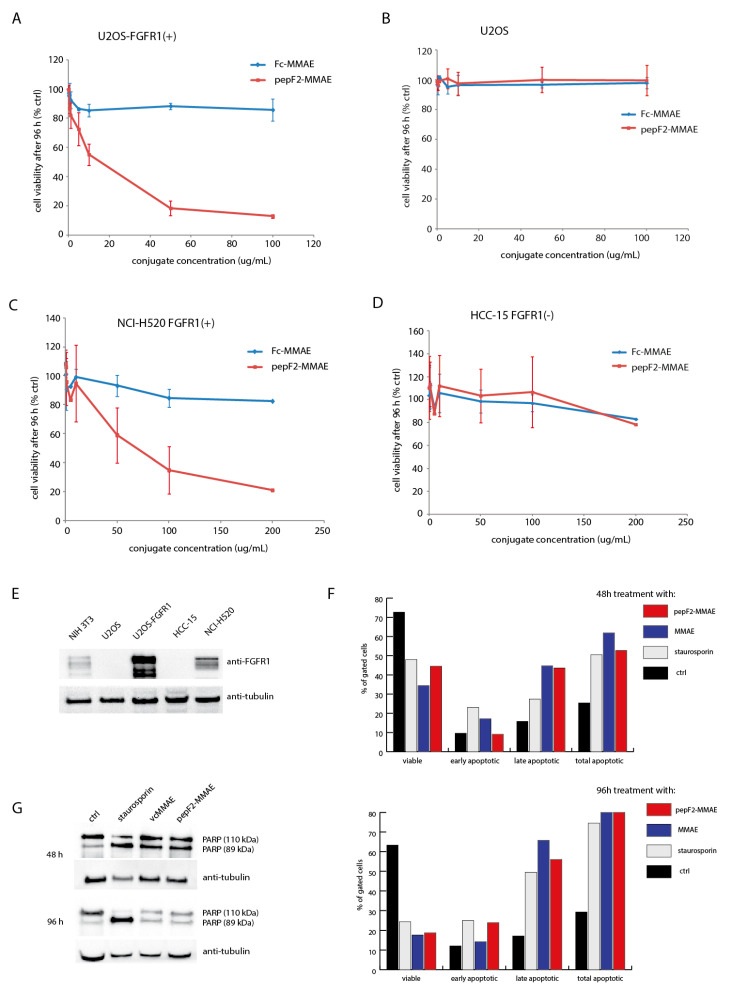
Cytotoxicity of peptibodyF2-MMAE and Fc-MMAE towards different cancer cell lines. (**A**,**B**)—Comparison of cytotoxic effects of peptibodyF2 and Fc conjugates on osteosarcoma cell lines with high (U2OS-FGFR1) and low (U2OS) levels of FGFR1. (**C**,**D**)—cytotoxic tests on lung cancer cell lines NCI-H520 expressing FGFR1 or FGFR1-negative HCC-15. All experiments were normalized to the values for non-treated cells recognized as 100%. The error bars represent ± SD for ***n*** = 3 experiments. (**E**)—FGFR1 levels in studied cell lines estimated by Western blot. (**F**,**G**)—Treatment with peptibodyF2-MMAE leads to cell apoptosis, similarly as for MMAE alone, as shown by FACS analysis with PI/Annexin V staining (**F**), and Western blot analysis of PARP cleavage (**G**).

Our approach relies on a peptibody serving as a delivery vehicle for the cytotoxic agent, so a crucial part of the study was to evaluate if specific internalization of peptibodyF2 translates to receptor-specific cytotoxicity once the peptibody is conjugated with MMAE. The cytotoxicity tests were performed using osteosarcoma cell lines (U2OS, U2OS-FGFR1) and non-small lung cancer cell lines (HCC-15, NCI-H520). First, we evaluated the cytotoxicity of peptibodyF2-MMAE on osteosarcoma cells with low expression level of FGFR1 (U2OS) and such cells stably transfected with ***fgfr1*** (U2OS-FGFR1). U2OS-FGFR1 cells proved to be sensitive for peptibodyF2-MMAE, while for U2OS we did not observe significant cytotoxic effect (Figure 6A,B). A similar cytotoxic effect of peptibodyF2-MMAE was obtained for non-modified, FGFR1-positive NCH-H520 lung cancer cells (Figure 6C). Furthermore, natural cell line devoid of FGFR1 expression, HCC15, was insensitive to peptibodyF2-MMAE (Figure 6D). In general, cytotoxicity of peptibodyF2-MMAE correlated with the FGFR1 expression level on tested cell lines. Cell lines sensitive to the conjugate showed also significantly higher expression levels than observed for fibroblast cells (Figure 6E).

To verify if the observed cytotoxic effect of peptibodyF2-MMAE was strictly dependent on the specific peptideF2–FGFR1 interaction and subsequent receptor-mediated uptake of peptibodyF2-MMAE the Fc domain conjugated to MMAE was used. Fc-MMAE did not decrease viability of either FGFR1-positive cell lines (U2OS-FGFR1 and NCI-H520) or FGFR1–negative cell lines (U2OS and HCC-15; Figure 6).

MMAE is a compound known to target microtubule polymerization and induce apoptosis [30]. Fluorescence-activated cell sorting (FACS) analysis with PI/Annexin V staining (Figure 6F) and Western blot analysis of PARP cleavage (Figure 6G) have shown that prolonged exposure of cells to either free MMAE or peptibodyF2-MMAE conjugate leads to an increase in the number of apoptotic cells. These results confirm that peptibodyF2-MMAE cytotoxicity is due to the presence of MMAE and occurs via induction of apoptosis.

These data demonstrate that peptibodyF2-MMAE was highly efficient and selective in recognizing FGFR1-expressing cells and triggering their death. The cytotoxicity assay results correlated well with internalization tests and show high selectivity of peptibodyF2 conjugate.

## 3. Discussion

Despite multiple antibody-based and antibody–drug conjugates (ADC)-based therapies studied in clinical trials, alternative targeting molecules are still being sought and tested. Combined selectivity of the targeting peptide and good pharmacokinetic properties of the Fc domain enable peptibodies to be considered as a promising alternative to monoclonal antibodies in targeted therapy [31]. The main advantage of peptide-Fc fusions is their size, which in the case of mAbs and therapeutic peptides can pose problems—when considering antibodies large molecular size may limit their efficient penetration of solid tumor tissue [32], whereas for peptides alone a small size causes their quick removal from the bloodstream and very short half-life [33,34,35,36]. Fusing to the Fc domain renders therapeutic peptides more stable, minimizes the immunological response and increases their affinity to the target due to the avidity effects [16].

The peptide described here serving as an FGFR-targeting portion of designed peptibodyF2 is a fragment of FGF2 (natural FGFR ligand) sequence. It is based on an observation that two previously independently reported FGFR-binding peptides based on FGF2 sequence are located in direct adjacency in the FGF2 sequence (region 48–58— [23] and region 59–68— [24]). We decided to link these two sequences together, potentially maximizing the region interacting with FGFR1 that is likely to exhibit higher affinity to the receptor compared to shorter peptides [37]. The resulting peptideF2 spans residues 48–68 of FGF2 (DPHIKLQLQAEERGVVSIKGV) and was expressed as a Fc-fusion named peptibodyF2.

Considering that peptibodyF2 contains FGF2 derived-peptide, it is possible that the peptibodyF2 binding site on the FGFR will be the same or similar as for the natural ligand. It may cause either beneficial competition with growth factors and reduced activation of targeted FGFRs [38,39], or act as an agonist and cause receptor activation [40]. Here, we observed the stimulation of FGFR-depended signaling pathways when cells were treated with peptibodyF2, albeit the activation was seen only at much higher concentrations of the peptibody in relation to FGF1 (300-fold more of peptibodyF2 is needed for a similar level of receptor activation) [41]. A much lower ability of peptibodyF2 to cause a cellular response is not surprising, as the binding affinity may be significantly lower than for the full length growth factors—the targeting peptide in the peptibody is a fraction of growth factor sequence and does not cover all receptor binding regions within the ligand. Moreover, peptibodyF2 did not show induction of a long-term cellular response—it did not stimulate proliferation of NIH 3T3 cells (data not shown), which is a desired feature of the targeting molecule for cancer treatment.

Receptor activation suggests a specific peptibodyF2–FGFR interaction. These findings have been further confirmed with peptibodyF2 cell uptake studies using fluorescence microscopy. PeptibodyF2 was internalized into FGFR1 overexpressing cells strictly via receptor-mediated endocytosis, suggesting that it may serve as a drug carrier for selective elimination of FGFR1-bearing cancer cells. Moreover, experiment based on adding increasing concentrations of FGF1 together with constant peptibody concentration indicated that the peptibody binding occurs in the same region of FGFR1 as growth factor binding, as the signal from the peptibody disappeared with increasing FGF1 concentration.

We subsequently generated a cytotoxic conjugate of peptibodyF2 and verified its potency for targeted delivery of the cytotoxic drug into FGFR1-overexpressing cells. Using the similarity of peptibodies to antibodies, we used the concept of ADC molecules to construct a peptibody conjugate with the drug. Therefore, we conjugated peptibodyF2 with monomethyl auristatin E (MMAE) using a strategy analogous to ADC [42]. This cytotoxic drug has been widely characterized due to its potent antimitogenic properties by blocking tubulin polymerization [43]. The potency of MMAE is 100–1000 times higher than doxorubicin and it cannot be used as a drug itself [44]. However, by attaching MMAE to monoclonal antibodies or fusion proteins with the Fc fragment, it has been successfully applied for site specific drug delivery, minimizing the cytotoxicity of the drug to healthy cells [45,46,47,48]. MMAE has been used in three drugs approved and on the market with multiple others following in clinical trials and has become one of the few widely used drugs for such applications.

According to our knowledge, this is the first time that a peptibody-type molecule was used as a carrier for a targeted delivery of cytotoxic drug. There has been a recent report of a “pseudo-antibody”, a knottin-Fc fusion with specific integrin binding properties, with monomethyl auristatin F (MMAF) cytotoxic drug attached to it [46]. However, knottins, which are mini-proteins with 3 disulphide bonds in their structure are not commonly perceived as short peptides used in the construction of peptibody-type molecules.

The developed peptibody-based conjugate is meant to be internalized after binding to their target on the cancer cell surface and release the cytotoxic drug inside the cells, leading to cell death. Cytotoxic potency was evaluated on FGFR1-positive cell lines—model engineered cell line (U2OS-FGFR1) and lung cancer cell line (NCI-H520). We showed that the conjugate exhibits specific cytotoxicity towards FGFR1-positive cell lines at micromolar concentrations of peptibodyF2-MMAE. Compared to other conjugates natural ligands with cytotoxic drug like FGF1-MMAE and FGF2-MMAE, a similar decrease in viability was observed at nanomolar concentrations [49,50]. FGF conjugates, although showing higher efficacy in eliminating FGFR-expressing cells, carry the risk of introducing additional growth factors into cancer regions, thus reducing the therapeutic effect. High potency of antibodies or antibody fragments conjugated with MMAE can be attributed to the larger antibody–antigen interface than in the case of peptide-based binders. Nevertheless, the molecule size is also important in drug delivery strategies—molecular weight of conjugates scFv-Fc-MMAE and knottin-Fc-MMAE [46,51] is two times higher than peptibodyF2 and may cause impeded penetration into solid tumors.

Based on the shown here specific cytotoxicity, peptibodyF2-MMAE emerges as an attractive alternative to larger targeting molecules used in the anticancer therapy. Features like selective cytotoxicity, relatively small size and high expression efficiency make peptibody-based conjugates promising candidates for future development.

## 4. Materials and Methods

### 4.1. Cell Lines

CHO-S cells were obtained from Thermo Fisher Scientific and cultured in PowerCHO medium (Lonza, Basel, Switzerland) supplemented with 8 mM L-glutamine (Lonza, Basel, Switzerland) and 1% penicillin-streptomycin mix (Biowest, Nuaillé, France) at 37 °C in a humidified incubator with 8% CO_2_. U2OS (human bone osteosarcoma epithelial cells), NIH 3T3 (mouse embryo fibroblasts), and NCI-H520 (lung squamous cell carcinoma, FGFR1-positive) were obtained from American Type Culture Collection (ATCC). U2OS cells stably expressing FGFR1 (U2OS-FGFR1) were a kind gift from Dr. Ellen M. Haugsten from the Norwegian Radium Hospital and HCC-15 (lung squamous cell carcinoma, FGFR1-negative) was from Leibniz Institute DSMZ—German Collection of Microorganisms and Cell Cultures. U2OS, NIH 3T3 were cultured in DMEM with 10% FBS and 1% penicillin-streptomycin mix (Biowest, Nuaillé, France), U2OS-FGFR1 was cultured in DMEM with 10% FBS, 1% penicillin-streptomycin mix (Biowest, Nuaillé, France) and 0.2 mg/mL geneticin (Thermo Fisher Scientific, Waltham, MA, USA). HCC-15 and NCI-H520 were cultured in RPMI 1640 (ATCC) with 10% FBS and 1% penicillin-streptomycin mix (Biowest, Nuaillé, France).

### 4.2. Expression and Purification of Peptibody and Fc Fragment

We used the numbering of FGF2 residues according to Facchiano et al., 2003 [24], therefore a sequence corresponding to residues 48–68 in FGF2 was used. The peptibodyF2 coding sequence was cloned into the pLEV113 vector and expressed in CHO-S cells (Chinese Hamster Ovary). The expression and purification were based on the protocol described previously for FGFR1-Fc fusion protein, with expression time increased to 10 days [25,51].

### 4.3. Activation of FGFR1-Dependent Pathways in NIH 3T3 Cells

The cells were seeded on 6-well plates at 2 × 10^5^ cells per well in the medium containing 10% FBS and after 24 h the medium was changed to the medium without serum. After 24 h serum starvation peptibodyF2 at increasing concentrations (0.5–20 µg/mL, equivalent to 1.6 × 10^−5^ M – 6.67 × 10^−4^ M) was added to the cells and incubated for 15 min, 24 h or 48 h at 37 °C in 5% CO_2_ and humidified atmosphere. As the controls FGF1 (10 ng/mL) and Fc (20 µg/mL) were added to the cells. For longer timepoints (24 h and 48 h) heparin was added to the medium to final concentration of 10 U/mL. The cells were lysed in 2 × SDS loading sample buffer (100 mM Tris-HCl pH 6.8, 4% SDS, 0.2% bromophenol blue, 200 mM β-mercaptoethanol, 20% glycerol), boiled and sonicated.

Cell lysates were resolved in 10% SDS-PAGE electrophoresis and then transferred onto the PVDF membrane (Millipore, Darmstadt, Germany). The following primary antibodies were used in Western blotting analysis: phospho-FGFR (Cell Signaling Technology, Danvers, MA, USA), phospho-p44/42 MAPK (p-Erk1/2; Cell Signaling Technology, Danvers, MA, USA), phospho-Akt (Thr308; Cell Signaling Technology, Danvers, MA, USA), Phospho-PLCγ1 (Tyr783; Cell Signaling Technology, Danvers, MA, USA), Phospho-FRS2-α (Tyr196; Cell Signaling Technology, Danvers, MA, USA) and Anti-γ-Tubulin antibody (Sigma Aldrich,). Detection was performed with HRP-conjugated secondary antibodies (Jackson ImmunoResearch, Cambridgeshire, UK).

### 4.4. FGF1-Induced Proliferative Response

On the 96-well plates 24 h serum-starved NIH 3T3 cells were treated with FGF1 (1, 10 and 100 ng/mL), peptibody F2 or Fc (0.5, 1, 10 and 20 µg/mL). After 48 h, cell viability was determined by the AlamarBlue cell viability reagent (Thermo Fisher Scientific, Waltham, MA, USA). Emission of fluorescent reduced form of the dye was measured at 590 nm upon excitation at 560 nm on the Infinite M1000 PRO plate reader (Tecan, Männedorf, Switzerland).

### 4.5. Fluorescence Microscopy

In all experiments using fluorescent microscopy U2OS-FGFR1 or U2OS cells were seeded at 1 × 10^4^ per each well on 96-well plate and incubated overnight at 37 °C in 5% CO_2_ and humidified atmosphere.

For analysis of internalization cells were starved for 16 h at 37 °C in 5% CO_2_ and humidified atmosphere. Next day, the medium was changed for the medium with addition of 1% BSA and the plate was cooled for 20–30 min. PeptibodyF2 (20 µg/mL) and Fc (20 µg/mL) were incubated with the cells at 4 °C for 15 min and then at 37 °C for 30 min. Cells were fixed with 4% paraformaldehyde, washed with PBS, permeabilized with 0.1% Triton-X100 (Biowest, Nuaillé, France) in PBS for 10 min and blocked with 2% BSA in PBS for 30 min. The Fc-bearing proteins were visualized with a fluorescent dye Zenon Alexa Fluor 488 Rabbit IgG Labeling Kit (Thermo Fisher Scientific, Waltham, MA, USA) following the manufacturer’s protocol. The cells were washed in PBS and fixed again with 4% paraformaldehyde. The nuclei were labeled with NucBlue Live (Thermo Fisher Scientific, Waltham, MA, USA) for 5 min. For colocalization analysis of internalized peptibodyF2 with early endosomes the cells were transfected with CellLight™ Early Endosomes-RFP, BacMam 2.0 (Thermo Fisher, Waltham, MA, USA) prior the addition of peptibodyF2. In competition experiments peptibodyF2 (20 µg/mL) was incubated with the cells at 37 °C for 30 min in the presence of different FGF1 concentrations (from 5 µg to 40 µg per well).

Wide-field fluorescence microscopy was performed with Zeiss Axio Observer Z1 fluorescence microscope using a LD-Plan-Neofluar 40/0.6 objective and Axiocam 503 (Zeiss, Oberkochen, Germany). The fluorescence of CellLight™ Early Endosomes-RFP was visualized with a 540/552 nm bandpass excitation filter and a 575/640 nm bandpass emission filter. The fluorescence of Zenon Alexa Fluor 488 was visualized with a 450/490 nm bandpass excitation filter and a 500/550 nm bandpass emission filter. Images were processed with Zeiss ZEN 2.3 software (Zeiss, Oberkochen, Germany), and Adobe Photoshop CS6 (Adobe, San Jose, CA, USA).

### 4.6. Conjugation of PeptibodyF2 and Fc Fragment with MMAE

To reduce disulfide bonds in the hinge region of Fc domain, proteins at the concentration of 1 mg/mL in 2 mL of PBS solution (100 mM NaCl, 18 mM NaH_2_PO_4_ and 33 mM Na_2_HPO_4_ pH 7.4) were reduced using reduction buffer (at a final concentration of 1 mM EDTA and 1 mM TCEP pH 6.0) for 1 h at 37 °C. 16-fold molar excess of vcMMAE (at stock concentration 100 µg/mL) (MedChemExpress, NJ, USA) over protein-SH groups was added. Next, double volume of PBS buffer relative to the volume of protein was added, and finally, the reduced protein solutions were added to the tubes with vcMMAE. Reduced proteins were incubated with the cytotoxic drug at 15 °C overnight with constant mixing. To confirm the efficiency of conjugation, SDS-PAGE electrophoresis was performed with the samples from the conjugated and mock-treated proteins.

PeptibodyF2-MMAE and Fc-MMAE were purified on HiTrap Protein A HP column (GE Healthcare, Upasala, Sweden). Column was washed with MilliQ and 10 CV (column volumes) of PBS (pH 7.4). After the conjugate was loaded onto the column, it was washed with PBS (5 CV) and backflow elution was carried out using 0.1 M sodium citrate, pH 3.0. To neutralize the low pH of the elution buffer 1 M Tris, pH 9.0 was added to the sample immediately after elution (in proportion 1 mL of protein to 200 μL of 1 M Tris pH 9.0). After purification the protein was dialyzed to PBS using Slide-A-Lyzer™ Dialysis Cassette (Thermo Fisher Scientific, Waltham, MA, USA).

### 4.7. Protein Stability in Cell-Conditioned Medium

PeptibodyF2 or peptibodyF2-MMAE was added to the U2OS-FGFR1 or NIH 3T3 cells seeded on 6-wells plates at 2 × 10^5^ cells per well, and incubated with the cells at 37 °C. After indicated times samples were taken, mixed with SDS sample buffer, boiled and frozen. Samples were separated by SDS-PAGE and analyzed by Western blotting with anti-Fc antibodies. The original Western blot figures can be found in the Supplementary File.

### 4.8. Cytotoxicity Assay

The cytotoxicity of peptibodyF2-MMAE and Fc-MMAE was tested on four cell lines (U2OS, U2OS-FGFR1, HCC-15 and NCI-H520). The cells were seeded on 96-well plates (10^4^ cells per each well) in appropriate medium with 10% FBS and antibiotics. After 24 h of incubation the conjugates were added to the cells (0.05–200 µg/mL, equivalent to 1.67 × 10^−8^–6.67 × 10^−6^ M). The plates were incubated at 37 °C in 5% CO_2_ and humidified atmosphere for 96 h. To measure the fluorescence level of dead cells Triton X-100 (Biowest, Nuaillé, France) was added to the non-treated cells to a final concentration of 0.1%. The cytotoxicity of tested conjugates was determined using Alamar Blue (Invitrogen, Waltham, MA, USA). The fluorescence intensity was measured with an excitation of 560 nm and an emission of 590 nm using microplate reader (Infinite M1000 pro, Tecan, Männedorf, Switzerland). Each sample dilution was prepared in triplicate and every experiment was repeated at least 3 times.

### 4.9. Annexin V-7AAD Assay

U2OS-FGFR1 cells (at the concentration 1 × 10^5^ per well) were seeded onto two 6-well plates and incubated overnight at 37 °C with 5% CO_2_ and humidified atmosphere. On the following day, the cells were treated with peptibodyF2-MMAE (10 µg/mL) and MMAE (1 nM) for 48 h or 96 h, respectively. As a control nontreated cells were used. Finally, the cells were collected with 10 mM EDTA, pH 8.0 and stained with Annexin V and 7AAD following with manufacturer’s protocol (Muse^®^ Annexin V and Dead Cell Kit, Merck Millipore, Burlington, MA, USA). Detection of apoptosis was carried out using Muse Cell Analyzer (Merck Millipore, Burlington, MA, USA). The data were analyzed with Muse 1.3.1 Analysis Software (Merck Millipore, Burlington, MA, USA).

### 4.10. PARP Cleavage

U2OS-FGFR1 cells were seeded onto two 6-well plates and incubated to allow it to adhere overnight. Cells were treated with peptibodyF2-MMAE and MMAE for 48 h or 96 h, respectively. As a control nontreated cells were used. Staurosporin (1 µM) was added to the additional wells 16 h before the end points and incubated at 37 °C with 5% CO_2_ and humidified atmosphere. Cells were lysed with 2 × SDS loading sample buffer (100 mM Tris-HCl pH 6.8, 4% SDS, 0.2% bromophenol blue, 200 mM β-mercaptoethanol and 20% glycerol), boiled and sonicated.

Samples were resolved in 10% SDS-PAGE electrophoresis and transferred onto the PVDF membrane (Millipore, Darmstadt, Germany). In this experiment PARP antibody (Cell Signaling Technology, Danvers, MA, USA) was used. Detection was performed with HRP-conjugated secondary antibodies (Jackson ImmunoResearch, Cambridgeshire, UK).

## 5. Conclusions

Peptibody based on the FGF2-derived peptide (peptide F2) binds to FGF receptors expressed on the cells and is specifically internalized in a receptor-dependent manner, rendering it a selective drug carrier. PeptibodyF2-MMAE, being peptibodyF2 conjugated with a cytotoxic drug, shows high and specific cytotoxicity towards cell lines expressing FGFR1, i.e., squamous cell lung carcinoma NCI-H520, while remaining non-toxic for cells with physiological FGFR1 levels. Developed here novel FGFR1-specific peptibody can serve as a basis for further advancement of clinically relevant FGFR1-targeted treatments.

## 6. Patents

A patent application no. P.433329, describing the use of peptibodyF2 conjugates for selective elimination of FGFR1-expressing cells, has been filed to The Patent Office of the Republic of Poland by the University of Wroclaw.

## Supplementary Materials

The following are available online at https://www.mdpi.com/2072-6694/12/10/2992/s1, Supplementary file: The original Western blot figures.
