# Supplementary material for: FGF2-Derived PeptibodyF2-MMAE Conjugate for Targeted Delivery of Cytotoxic Drugs into Cancer Cells Overexpressing FGFR1
Source: Cancers (Basel). 2020 Oct 15;12(10):2992. doi: 10.3390/cancers12102992 (PMC7602595; doi:10.3390/cancers12102992)

Sup. Fig. 1 - Raw images for NIH 3T3 signaling Western blots after incubation with peptibodyF2 or Fc.
Order of samples as described on Fig. 3A.

- anti-phospho FGFR1 Western blot:

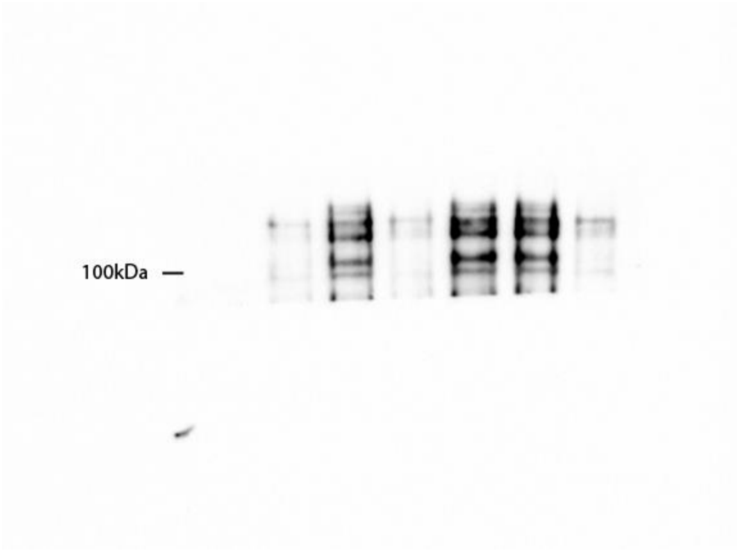

- anti-phospho Akt1 Western blot:

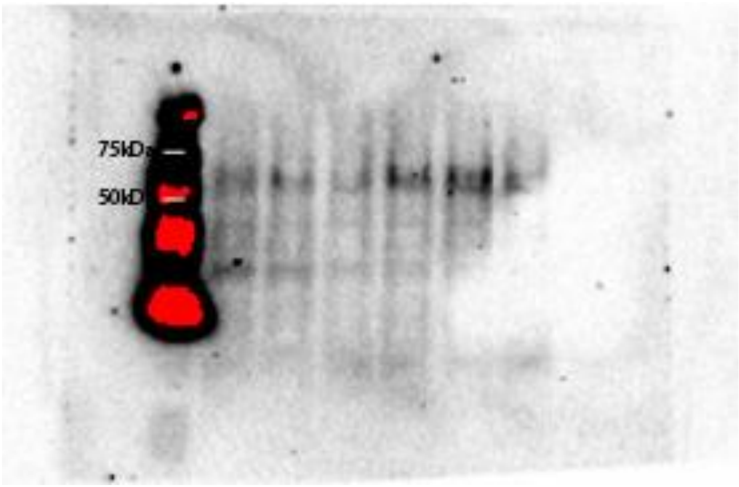

- anti-phospho PLCgamma Western blot:

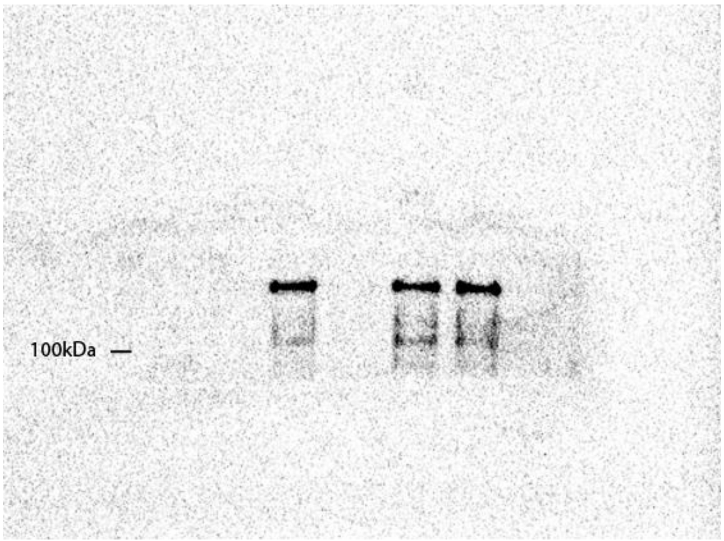

- anti-phospho FRS2 Western blot:

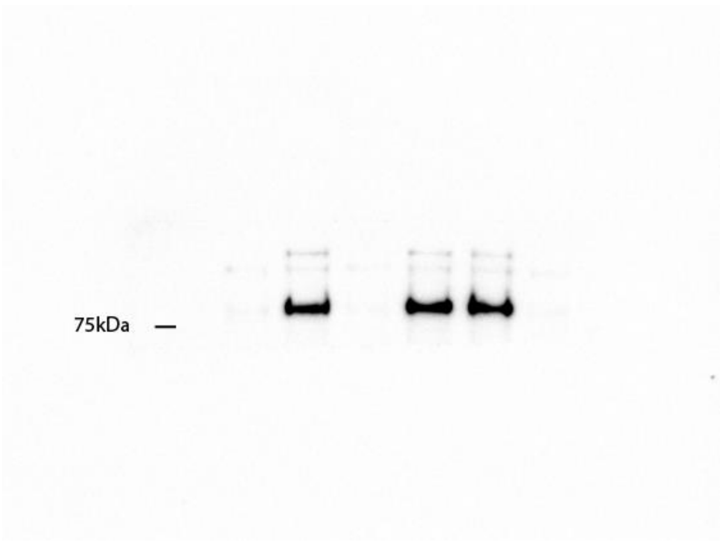

- anti-phospho Erk1,2 Western blot:

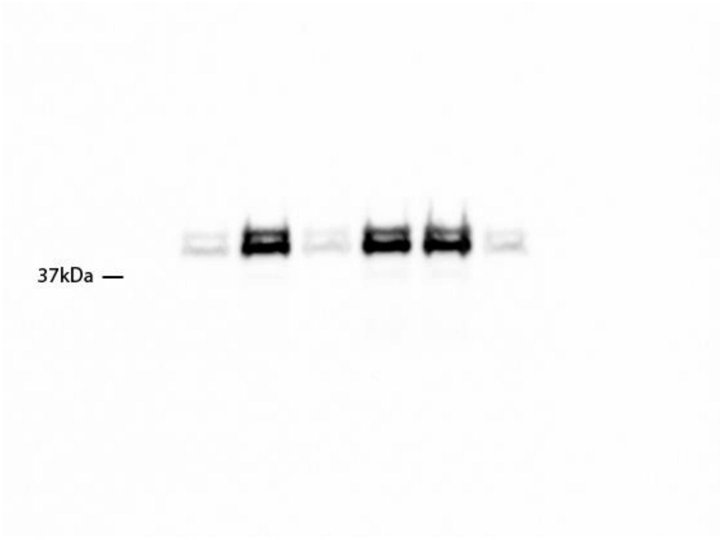

- anti-tubulin Western blot:

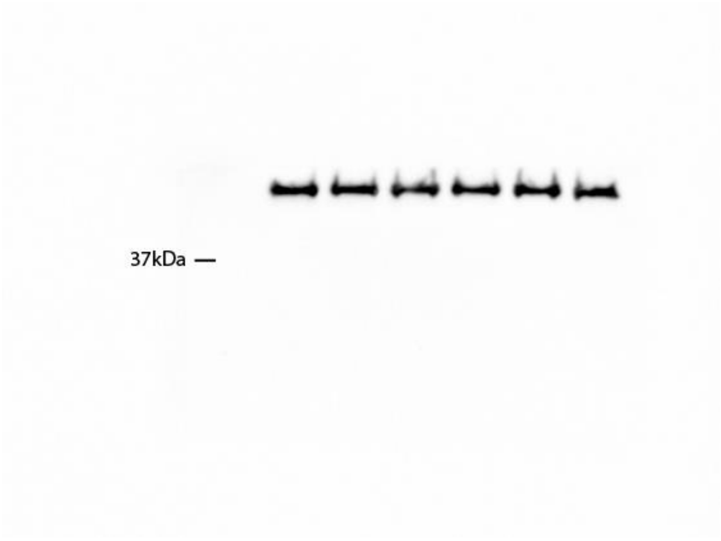

Sup. Fig. 2 - Raw images for NIH 3T3 signaling Western blots after prolonged incubation with peptibodyF2. Order of samples as described on Fig. 3C.

- anti-phospho FGFR1 Western blot:

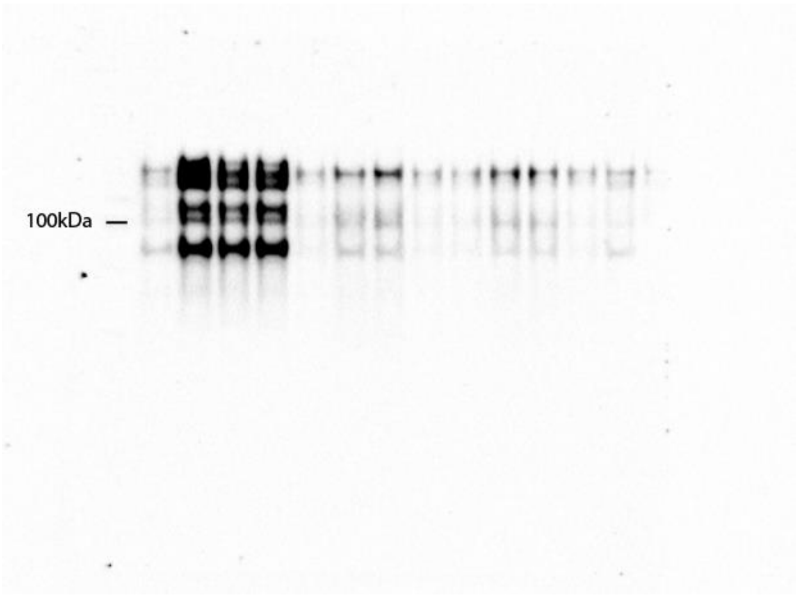

- anti-phospho Erk1,2 Western blot:

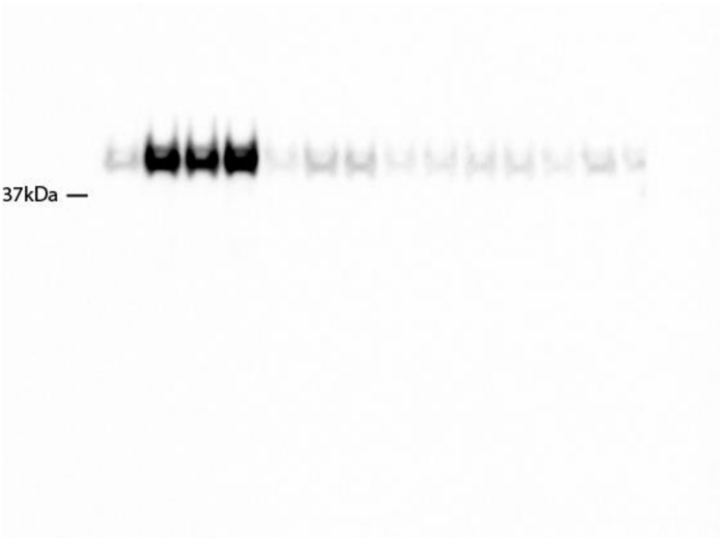

- anti-tubulin Western blot:

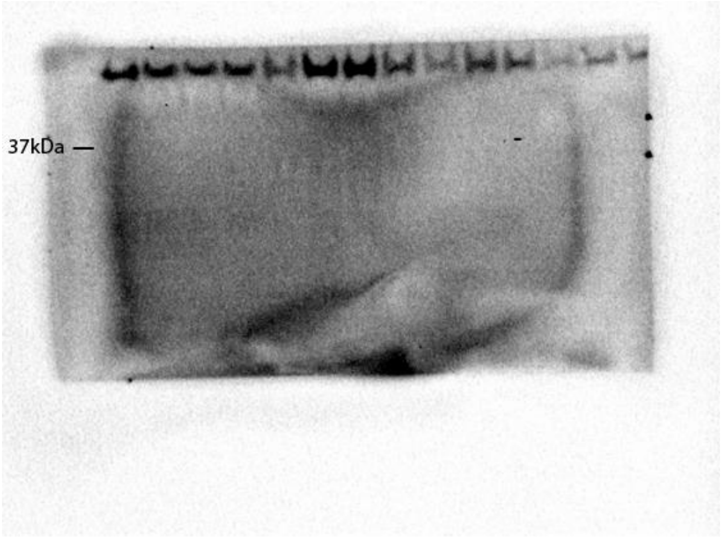

Sup. Fig. 3 - Raw images for anti-Fc Western blot detection of the amount of peptibodyF2 or peptibodyF2-MMAE conjugate present in the medium above U2OS-FGFR1 and NIH 3T3 cells. Order of samples as described on Fig. 5C.

- U2OS-FGFR1 cells, peptibodyF2:

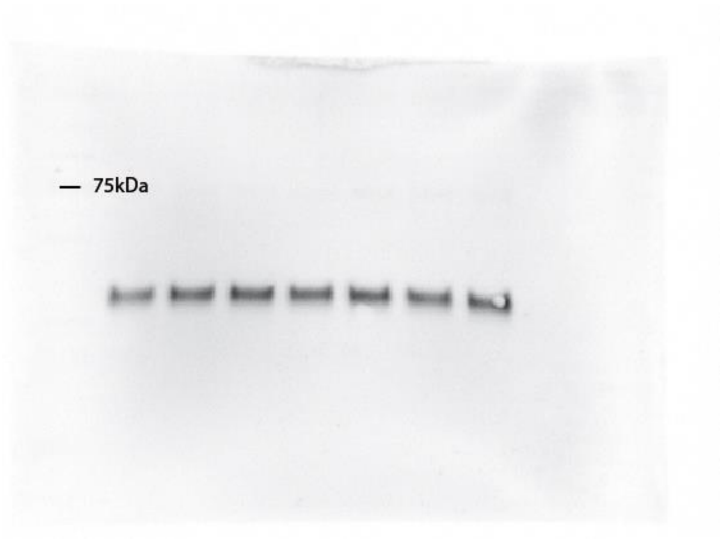

- U2OS-FGFR1 cells, peptibodyF2-MMAE:

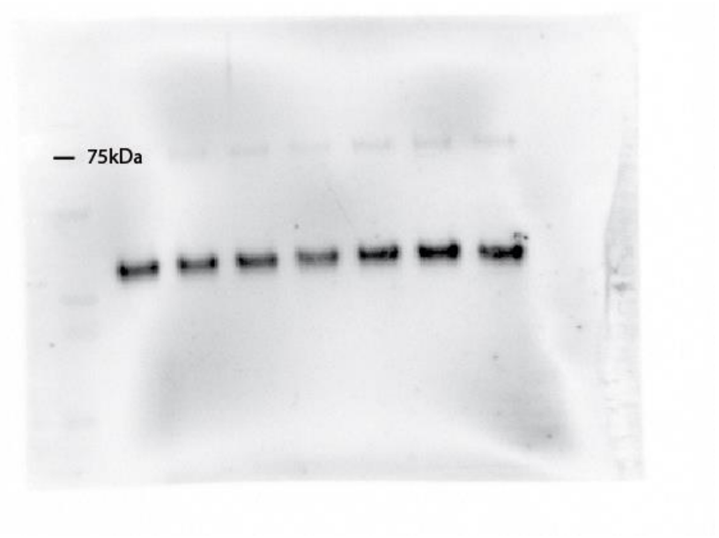

- NIH 3T3 cells, peptibodyF2:

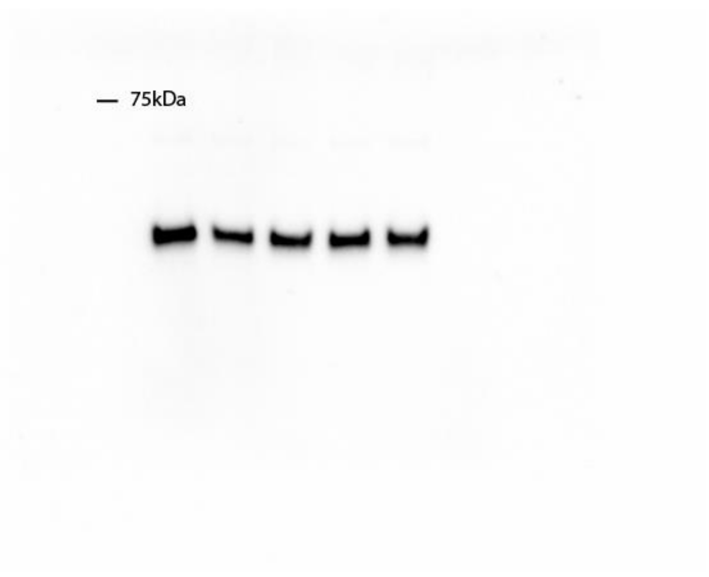

- NIH 3T3 cells, peptibodyF2-MMAE:

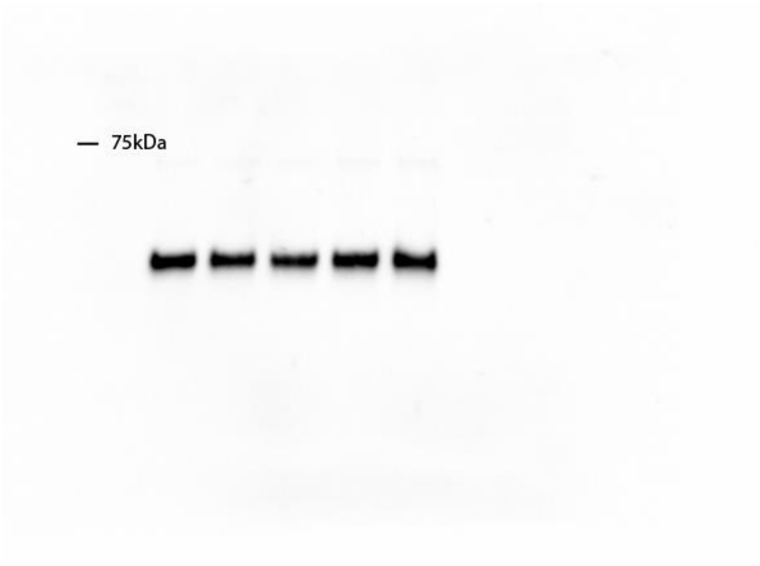

Sup. Fig. 4 - FGFR1 levels in studied cell lines estimated by Western blot. Order of samples as described on Fig. 6E.

- anti-phospho FGFR1 Western blot:

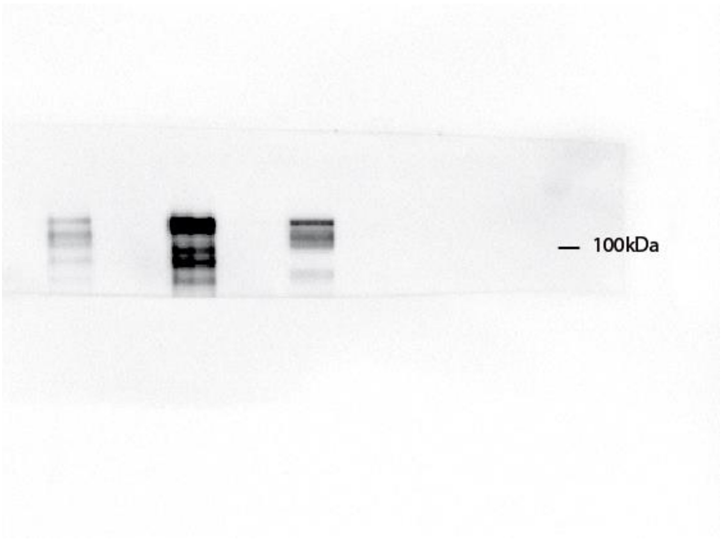

- anti-tubulin Western blot:

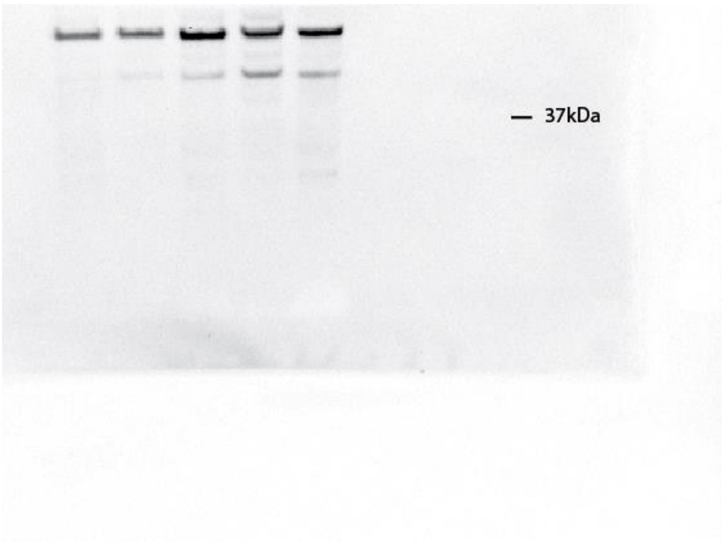

Sup. Fig. 5 - PARP cleavage induced by peptibodyF2-MMAE. Order of samples as described on Fig. 6G, additional one sample is peptibodyF2 replicate.

- anti-PARP Western blot, 48h:

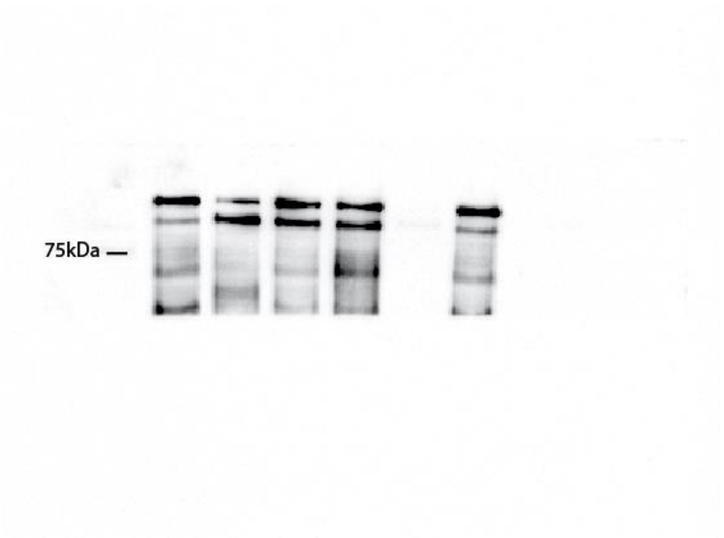

- anti-tubulin Western blot, 48h:

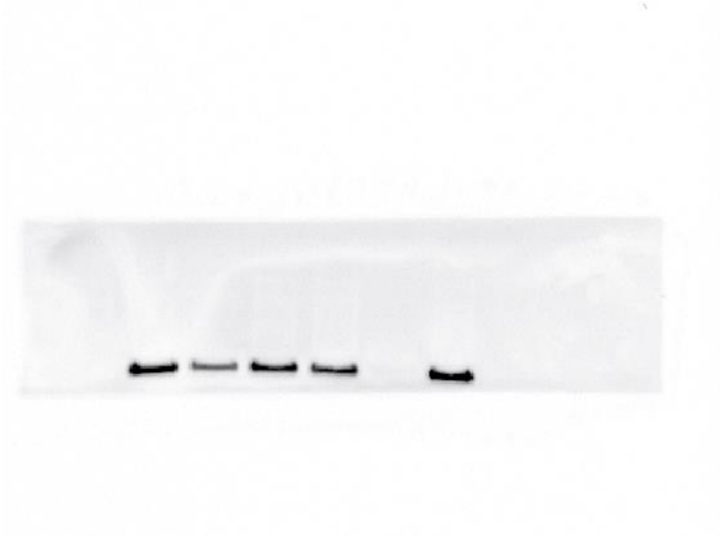

- anti-PARP Western blot, 96h:

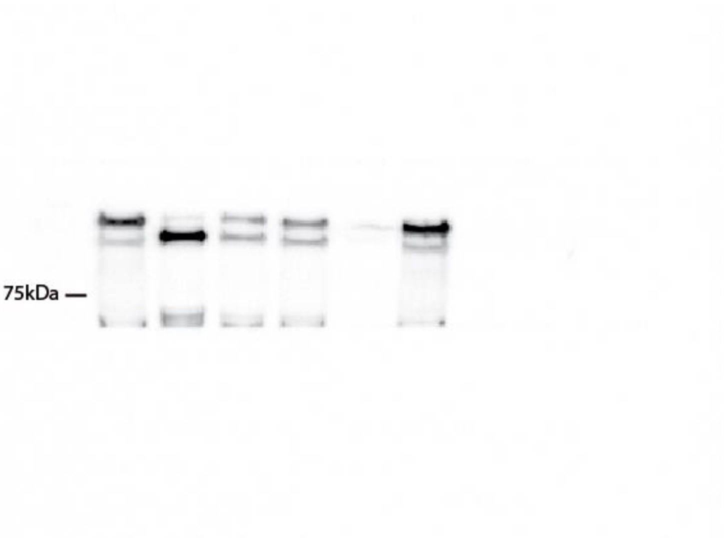

- anti-tubulin Western blot, 96h:

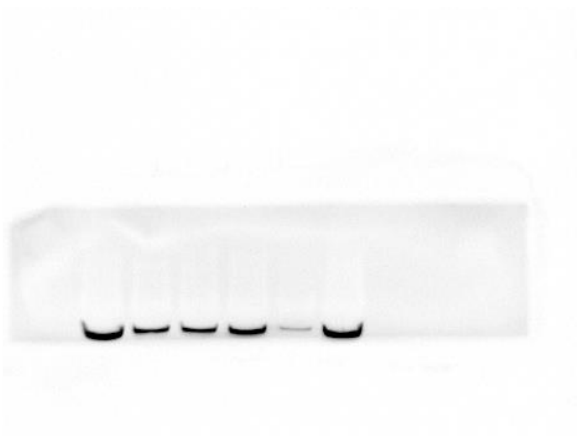

Supplement: Supplementary file 1 [file cancers-12-02992-s001.pdf]
